# SIMPLIFIED TECHNIQUE FOR RECONSTRUCTION OF THE DIGESTIVE TRACT AFTER TOTAL AND SUBTOTAL GASTRECTOMY FOR GASTRIC CANCER

**DOI:** 10.1590/S0102-67202014000200010

**Published:** 2014

**Authors:** Bruno ZILBERSTEIN, Carlos Eduardo JACOB, Leandro Cardoso BARCHI, Osmar Kenji YAGI, Ulysses RIBEIRO-JR, Brian Guilherme Monteiro Marta COIMBRA, Ivan CECCONELLO

**Affiliations:** Department of Gastroenterology, Digestive Surgery and Coloproctology Divisions, Faculty of Medicine, University of São Paulo, São Paulo, SP, Brazil.

**Keywords:** Gastrointestinal tract, Gastrectomy, Stomach neoplasms

## Abstract

**Background:**

Laparoscopic surgery has been increasingly applied to gastric cancer surgery. Gastrointestinal tract reconstruction totally done by laparoscopy also has been a challenge for those who developed this procedure.

**Aim:**

To describe simplified reconstruction after total or subtotal gastrectomy for gastric cancer by laparoscopy and the results of its application in a series of cases.

**Methods:**

In the last four years, 75 patients were operated with gastric cancer and two with GIST. Thirty-four were women and 43 men. The age ranged from 38 to 77 years with an average of 55 years. In two patients with GIST a total and a subtotal gastrectomy were performed. In the other 75 patients were done 21 total gastrectomies and 54 subtotal. In all cancers, gastrectomy with D2 lymphadenectomy was completed with at least 37 lymph nodes removed. Was used in these operations a modified laparoscopic technique proposed by the authors consisting in a latero lateral esophagojejunal anastomosis with linear stapler in TG as well in STG, and reconstruction of the digestive continuity also in the upper abdomen.

**Results:**

The intraoperative and immediate postoperative course were uneventful, except for one case of bleeding due to an opening clip, necessitating re-intervention. The operative time was 300 minutes, with no difference between total or subtotal gastrectomy. The number of lymph nodes removed varied from 28 to 69, averaging 37. Postoperative staging showed one case in T4 N2 M0; 13 in T2 N0 MO; 27 in T2 N1 M0; 24 in T3 N1 M0 and 10 in T3 N2 M0. Complication in only one case was observed on the 10th postoperative day with a small anastomotic leakage in esophagojejunal anastomose with spontaneous closure.

**Conclusion:**

The patient's evolution with no complications, no mortality and just one small anastomotic leakage with no systemic repercussions is a strong indication of the liability and feasibility of this innovative technical method.

## Abstract (pt)

**Racional:**

Cada vez mais a cirurgia laparoscópica está sendo aplicada no tratamento cirúrgico do câncer gástrico. A reconstrução do trato gastrointestinal totalmente feita por laparoscopia também tem sido um desafio para aqueles que desenvolveram este procedimento.

**Objetivo:**

Descrever reconstrução simplificada após gastrectomia total ou subtotal para o câncer gástrico por laparoscopia e os resultados de sua aplicação em uma série de casos

**Métodos:**

Nos últimos quatro anos, 75 pacientes foram operados com câncer gástrico e dois com GIST. Trinta e quatro eram mulheres e 43 homens. A idade variou de 38 a 77 anos com média de 55 anos. Nos dois pacientes com GIST em um foi realizada gastrectomia total e no outro gastrectomia subtotal. Nos outros 75 pacientes foram aplicados 21 gastrectomias totais e 54 subtotais. Em todos os cânceres, a gastrectomia foi completada com linfadenectomia D2 com pelo menos 37 linfonodos retirados. Foi utilizada nas operações modificação técnica laparoscópica proposta pelos autores consistindo em anastomose latero-lateral esôfago jejunal em GT e de modo semelhante na GST e restabelecimento da continuidade digestiva também no abdômen superior.

**Resultados:**

O intra-operatório e a evolução pós-operatória imediata transcorreu sem intercorrências, exceto por um caso de sangramento devido à abertura de clipe, sendo necessária re-intervenção. O tempo operatório foi de cerca de 300 minutos, não havendo diferença entre GT e GST. O número de nódulos linfáticos retirados variou de 28 a 69, com média de 37. O estadiamento pós-operatório mostrou um caso em T4 N2 M0; 13 em T2 N0 MO; 27 em T2 N1 M0; 24 em T3 N1 M0; e 10 em T3 N2 M0. Como complicação, em apenas um caso foi observado no 10º dia do pós-operatório uma pequena fístula da anastomose esofagojejunal com fechamento espontâneo.

**Conclusão:**

A evolução dos pacientes, sem complicações e mortalidade e com apenas um pequeno vazamento de anastomose sem repercussões sistêmicas, é forte indício da viabilidade técnica inovadora deste método.

## INTRODUCTION

Laparoscopic surgery has been increasingly applied to gastric cancer surgery. Gastrointestinal tract reconstruction totally done by laparoscopy also has been a challenge for those who developed this procedure. After introduction of laparoscopic stapling methods, the reconstruction techniques became more feasible. Different methods have been employed using circular or linear staplers^3,5,7,9,11,12^. On the other hand, in the last two decades there was also a great improvement in laparoscopic bariatric surgery with the performance of ingeniously developed techniques mainly for the reconstruction of digestive continuity.

Therefore, the aim of this study is to describe a simplified reconstruction technique after total or subtotal gastrectomy by laparoscopy for gastric cancer and the results of its application in a series of cases.

## METHODS

### Patients

In the last four years, there were operated 75 patients with gastric cancer and two patients with GIST. Thirty four were women and 43 men. The age varied from 38 to 77 years old with mean of 55 y. In the two patients with GIST it was performed one total (TG) and one subtotal gastrectomy (STG). In the other 75 patients were applied 21 TG and 54 STG. In all cancer patients, gastrectomy was completed by D2 lymphadenectomy with at least 37 lymph nodes removed.

### Operative technique

**Figure 1:**
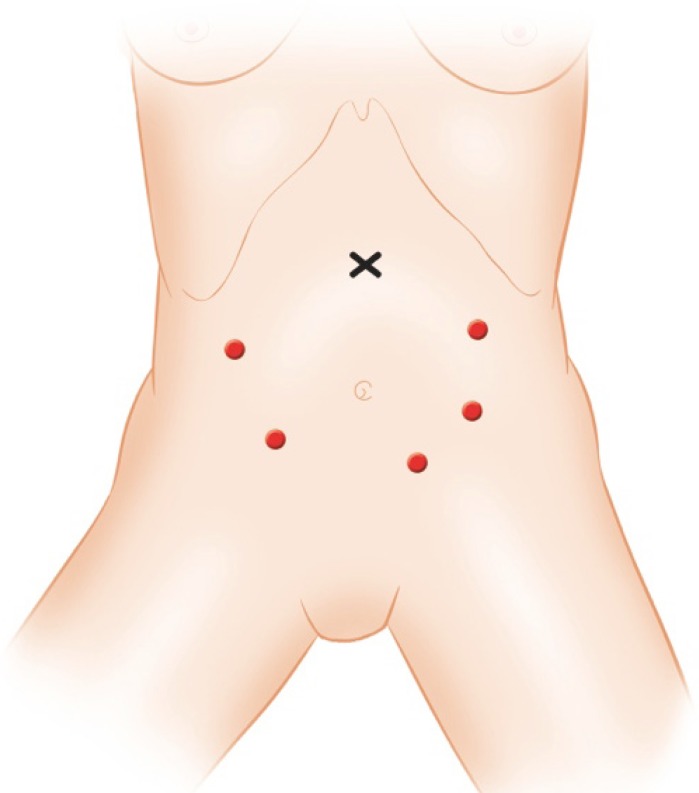
Port distribution on abdominal wall

After the induction of general anesthesia, the patient was placed in a modified lithotomy position, with the legs apart and flexed slightly. The surgeon stood between the legs, the first assistant (camera operator) on the patient's left and second assistant to the right side. After pneumoperitoneum was established, six ports were placed, consisting of two bilateral subcostal 5 mm ports and bilateral low abdominal 12 mm ports. Additional one of 10 mm was placed 5 cm below and 5 cm to the left of the patient for the camera. Finally, a 5 mm port was placed in the subxiphoidal space (Figure 1). Intracorporeal pressure was maintained at 12 to 15 mmHg.

The gastrectomy began with the mobilization of the greater curvature along the transverse colon, carried out with ultrasonic shears (laparoscopic coagulating shears LCS, Ethicon Endo-Surgery, Cincinnati, OH, USA). The roots of the right gastroepiploic and gastric vessels were exposed by delicate dissection. The infra-pyloric lymph nodes (LN station nº 6) were dissected and the vessels better exposed and, then, divided with double clips. During this exposure, occasionally, lymph nodes of LN station nº 14 can be removed. Strategically after this dissection, the dissection was moved through the hepatobiliary ligament, dividing the short vessels through the duodenum and liberating them just under the pylorus to facilitate the introduction of a 45 mm endoscopic linear stapler for the duodenal transection. Routinely, the reinforcement of the linear stapler was done with separated or continuous seromuscular suture. After, the dissection of the right gastric (pyloric) vessels was performed dividing them with clips and removing 12a LN station, then going through the common hepatic artery with the 8a LN station removal. By pushing-up the stomach, the left gastric vessels were easily identified. Also, during this maneuver, the pancreatic capsule could be removed if necessary. With the harmonic scalpel, the left gastric vessels was gently dissected and LN station nº 9 and 7 were removed. Dissection continued through the systematic dissection of the splenic artery toward the splenic hilum, removing LN station nº 11p, and 11d when necessary. This whole dissection is usually performed for D2 dissection in both TG and STG.

### Total gastrectomy 

When a TG was necessary, lymph node dissection continued dividing the peritoneal membrane that covers the esophagogastric junction and after exposing and tractioning the esophagus with a Penrose drain, vagal nerves were divided. This was followed by lymphnodal dissection of station 1, leaving in the gastric specimen lymph nodes of nº 3 station, and liberating the soft tissues on the posterior gastric wall. Then after, the greater curvature was moved again to finishing and releasing the great omentum from the transverse colon, moving through the left gastroepiploic vessels, dividing them with clips and then going through the short gastric vessels, which were divided with harmonic scalpel and clips when necessary.

**Figure 2:**
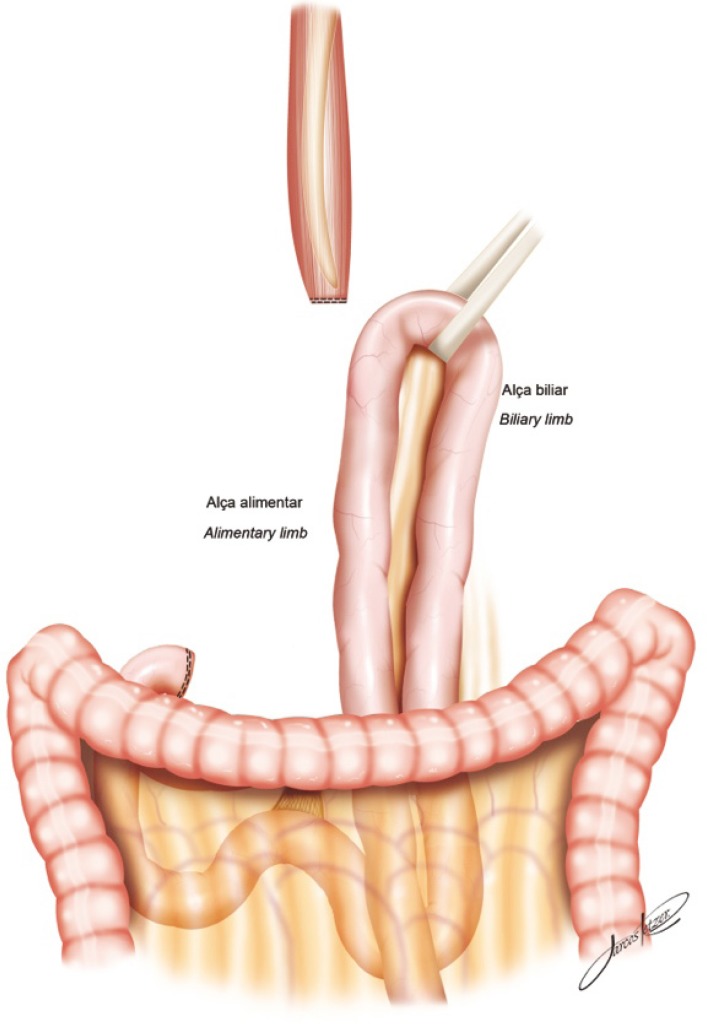
Pulling-up the alimentary and biliary jejunal limb to the supramesocolic space

**Figure 3:**
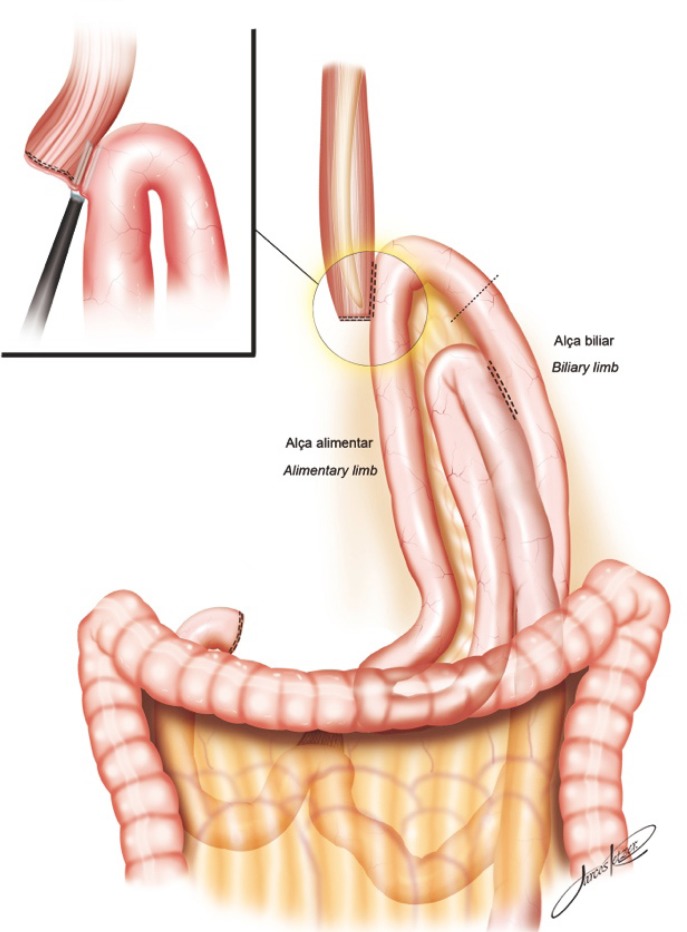
Esophajejunal anastomosis made with linear stapler and then the enteroenteral anastomosis of the biliary and alimentary limb, always in the upper abdomen

After completion of lymph node dissection, the esophagus was transected with a 45 mm linear stapler with blue or white cartridge, finishing the gastric resection. Continuity of digestive tract was performed with a Roux-en-Y diversion, totally done by laparoscopy. To facilitate this maneuver, the duodenojejunal angle was identified and a jejunal loop about 30-40 cm away was transposed to the supramesocolic space using transmesocolic or precolic route. The jejunal loop was anchored by a stitch to the left lateral wall of the abdominal transected esophagus (Figure 2). A 12 French bougie was orally introduced by the anesthesiologist to better expose the esophageal stump. A 45 mm linear stapler with white cartridge was utilized to perform the laterolateral esophagojejunal anastomosis (Figure 3). After the completion of the mechanical anastomosis, the bougie already exposing the esophagus was then introduced into the jejunal loop to ensure the anastomosis diameter and facilitate the closure of the common entry hole by hand sewn with extramucosal 3-0 PDS.

**Figure 4:**
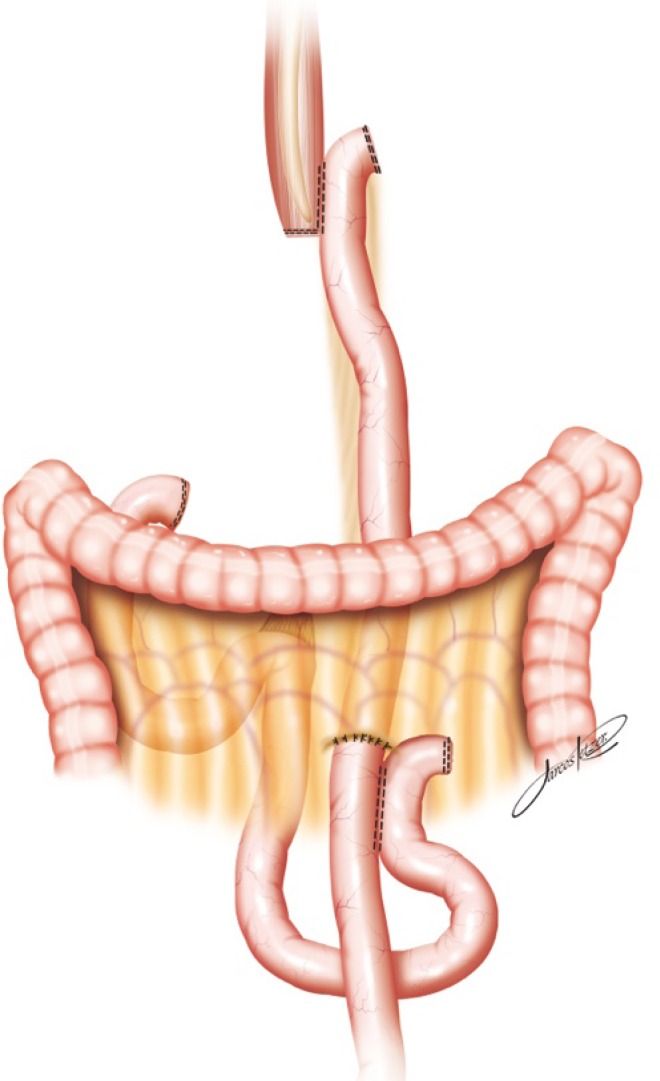
Final aspect of the reconstruction of the digestive continuity with Roux-en-Y diversion, after total gastrectomy

Next, the alimentary limb was isolated with about 70 cm long and also brought in the upper abdomen, close to the biliary limb in order to perform the jejunojejunal anastomosis of the Roux-en-Y reconstruction. This anastomosis was also performed with a 45 mm white cartridge linear stapler closing the stapler entrance by hand sewn with extramucosal 3-0 PDS®. Finished all the anastomosis, the closures were tested instilling by the esophageal bougie diluted methylene blue solution to ensure the good closure of the anastomosis. Once tested, the biliary limb and the alimentary limb were divided and separated by linear stapler with 45 mm white cartridge. In the transmesocolic route, the alimentary limb was tractioned and the enteroenteric anastomosis located in the inframesocolic space. The mesenteric gap was closed with manual interrupted suture (Figure 4).

### Subtotal gastrectomy

When subtotal gastrectomy was indicated, dissection of lymph node stations continued with the removal of the LN nº 1 station, leaving in the gastric specimen lymph nodes of nº 3 station. The greater curvature was moved again to finish and release the great omentum from the transverse colon, moving through the left gastroepiploic vessels, dividing them with clips. Depending on the gastric margins, the stomach was transected generally at that level with 60 mm linear stapler with one or two blue cartridges.

Continuity of digestive tract was performed also with a Roux-en-Y diversion totally done by laparoscopy. The duodenojejunal angle was identified and a jejunal loop about 30-40 cm away was transposed to the supramesocolic space using transmesocolic or precolic route. The jejunal loop was anchored by a stitch to the right lateral wall of the gastric stump. A gastrojejunal anastomosis was performed using a 60 mm linear stapler with blue cartridge. The common entry hole was then closed by hand sewn with extramucosal 3-0 PDS®.

**Figure 5:**
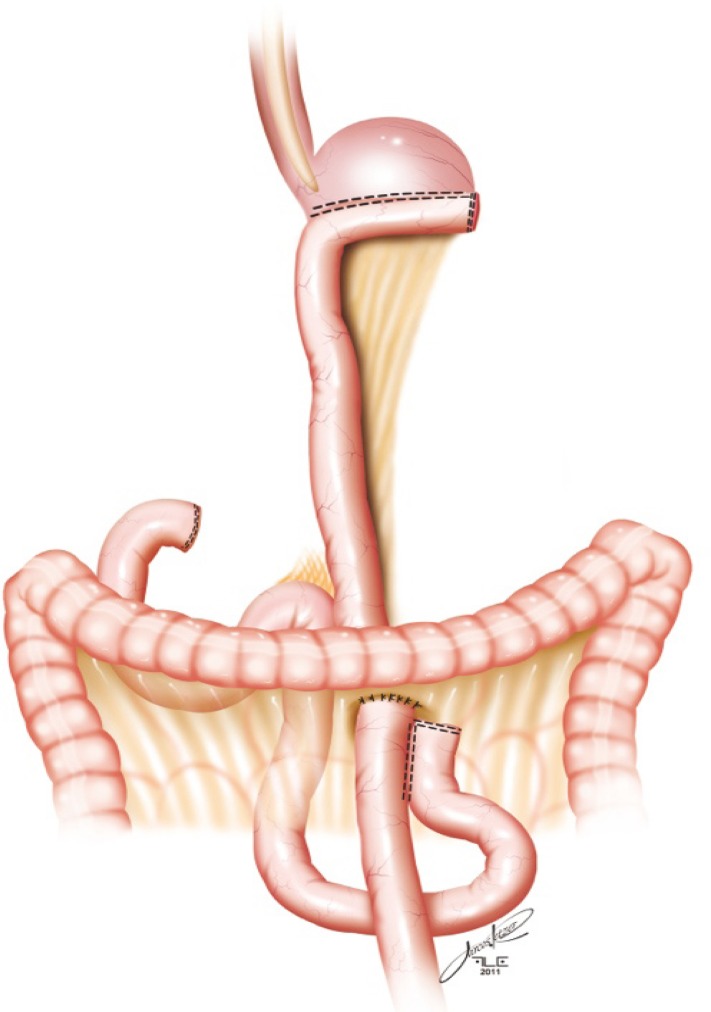
Final aspect of the reconstruction of the digestive continuity with Roux-en-Y diversion, after subtotal gastrectomy

Next, the alimentary limb was isolated with about 70 cm long and also brought in the upper abdomen, close to the biliary limb in order to perform the jejunojejunal anastomosis of the Roux-en-Y reconstruction. This anastomosis was performed with a 45 mm white cartridge linear stapler closing the stapler entrance by hand sewn with extramucosal 3-0 PDS®. The closures were tested instilling by a nasogastric tube diluted methylene blue solution to ensure the good closure of the anastomoses. Once tested, the billiary limb and the alimentary limb were separated utilizing linear stapler with 45 mm white cartridge. In the transmesocolic route, the alimentary limb was tractioned and the enteroenteric anastomosis was located in the inframesocolic space. The mesenteric gap was closed with interrupted suture (Figure 5).

### Finishing the operation and extracting the specimen

All the patients were drained with two silicon abdominal drains exteriorized by the lateral 12 mm ports, monitoring the esophagus or gastrojejunal anastomosis at the left and the duodenal stump, at the right. Usually, as the gastric specimen is large due to the amplified dissection and also great amount of grease of the greater omentum, for cosmetical reasons, a small Pfannenstiel suprapubic incision was made in order to remove the stomach.

## RESULTS

The intraoperative and immediate postoperative evolution was uneventful except for one case of bleeding due to clip escape where re-intervention was needed. The operative length was about 300 minutes, with no difference between TG and STG. Blood loss was minimal with no need of transfusion neither during nor after operation. The number of lymph nodes removed varied from 28 to 69 with a mean of 37.

Postoperative staging showed one T4 N2 M0 case (a young 38 y woman); 13 T2 N0 MO; 27 T2 N1 M0; 24 T3 N1 M0 and 10 T3 N2 M0.

All the patients were sent to intensive care unit where they remained for no more than 12 hours. In the first 24 hours, all patients were able to walk by themselves. The oral food intake was achieved in the 3^rd^ postoperative day for STG. In TG patients, enteral feeding was started also in the 3^rd^ postoperative day and lasted until the 10^th^ day, when a blue-methylene test was performed to confirm the integrity of the esophagojejunal anastomosis. Then, in the absence of anastomotic leakage, oral feeding was started. The abdominal drains were removed 48 hours after oral feeding had began. In just one case, was observed in the 10^th^ postoperative day a small leakage of the esophagojejunal anastomosis with spontaneous closure after 50 days.

The patients were discharged in the 7^th^ postoperative day in STG and usually in the 13^th^ in TG. Long-term follow-up showed good quality of life and normal food intake in all patients.

## DISCUSSION

Laparoscopic surgery for gastric cancer was and still is a very controversial applying technique. In oncological surgery survival is the goal. Therefore, cosmetic reasons cannot justify a less invasive technique unless it could promote lower complication, less pain, better recovery and, for sure, at least the same long term survival as conventional procedures.

Since 1992, when Goh et al.^2^ performed the first laparoscopic gastrectomy followed by Kitano et al. in 1994^4^ with the first laparoscopic gastrectomy for gastric cancer, countless publications showed the feasibility of this operation. Laparoscopic procedures for early gastric cancer rapidly gained acceptance and progress advances brought us even more developed techniques as robotic surgery^8^. Nevertheless, there is still a large discussion in utilizing these procedures for advanced gastric cancer and also due to the reconstruction techniques where mostly done by assisted method^11^.

Although the laparoscopic dissection and adequate lymphonodal removal can be achieved by this way, as demonstrated in this study with a mean number of 37 lymph nodes removed - demonstrating the feasibility of an acceptable D2 dissection -, the aim of this study was mostly to demonstrate an easy, simple, cheap and effective method of reconstruction of the digestive tract in TG or STG totally perform by laparoscopy, with all the advantages of a minimally invasive procedure^12^.

Laparoscopic access and dissection technique were well established^6,9,10^ and nowadays there is a strong tendency to utilize the laparoscopic or robotic procedure with this intention.

Nevertheless, reconstruction is still a problem and a lot of procedures were recommended each one with its impairments or difficulties, leading to the assisted method utilized for most surgeons.

In parallel with laparoscopic gastric surgery, there was a great development of bariatric surgery in occidental countries mainly in USA^1^. In the last decade, the reconstruction method for laparoscopic gastric bypass improved significantly with simple, fast and efficient techniques^13^. The current surgical method here proposed to reconstruct digestive tract after TG or STG totally by laparoscopy is a personal modification of a well established procedure adopted to reconstruct digestive tract after laparoscopic bypass and, also, advocated by other surgeons dedicated to gastric surgery^5,9^. The standardization of surgical steps allowing the surgeon to work comfortably in the same position between patient's legs, with the operative structures just in front of him, allows an ergonomic performance and, therefore, facilitates the operation giving the necessary insurance to achieve a reliable reconstructive method. Also, there is a very good exposure to perform the jejunojejunal anastomosis in the upper abdomen with the facilities already presented.

It has to be mentioned that the adoption of the same laparoscopic linear stapler for resection and also for reconstruction using in at most six cartridges to remove the stomach and to reconstruct digestive tract, is a very economical proposal. This method avoids the need of circular stapler^3^ and also obviates the difficulties and complications associated with the introduction of the anvil of the circular stapler through the mouth and the pharynx or sometimes its introduction through the abdominal wall.

## CONCLUSION

The patient's evolution with no complications, no mortality and just one small anastomotic leakage with no systemic repercussions is a strong indication of the liability and feasibility of this innovative technical method.
